# Identification of Genomic Regions for Deep-Water Resistance in Rice for Efficient Weed Control with Reduced Herbicide Use

**DOI:** 10.1186/s12284-023-00671-y

**Published:** 2023-11-25

**Authors:** Marina Iwasa, Koki Chigira, Tomohiro Nomura, Shunsuke Adachi, Hidenori Asami, Tetsuya Nakamura, Takashi Motobayashi, Taiichiro Ookawa

**Affiliations:** 1 Graduate School of Agriculture, Tokyo University of Agriculture and Technology, 3-5-8 Saiwai-cho, Fuchu, Tokyo 183-8509 Japan; 2 NARO Western Region Agricultural Research Center, 6-12-1 Nishifukatsu-cho, Fukuyama, Hiroshima 721-8514 Japan; 3 Yukimai Design Co. Ltd, 2-24-16 Nakamachi, Koganei, Tokyo 184-0012 Japan

**Keywords:** Deep-water resistance, GWAS, *NAL1*, *OsGA20ox1*, Reduced use of herbicides, Rice, Weed suppression

## Abstract

Deep-water (DW) management in rice fields is a promising technique for efficient control of paddy weeds with reduced herbicide use. Maintaining a water depth of 10–20 cm for several weeks can largely suppress the weed growth, though it also inhibits rice growth because the DW management is usually initiated immediately after transplanting. Improving the DW resistance of rice during the initial growth stage is essential to avoid suppressing growth. In this study, we demonstrate a large genetic variation in the above-ground biomass (AGB) after the end of DW management among 165 temperate *japonica* varieties developed in Japan. Because the AGB closely correlated with plant length (PL) and tiller number (TN) at the early growth stage, we analyzed genomic regions associated with PL and TN by conducting a genome-wide association study. For PL, a major peak was detected on chromosome 3 (*qPL3*), which includes a gene encoding gibberellin biosynthesis, *OsGA20ox1*. The rice varieties with increased PL had a higher expression level of *OsGA20ox1* as reported previously. For TN, a major peak was detected on chromosome 4 (*qTN4*), which includes *NAL1* gene associated with leaf morphological development and panicle number. Although there was less difference in the expression level of *NAL1* between genotypes, our findings suggest that an amino acid substitution in the exon region is responsible for the phenotypic changes. We also found that the rice varieties having alternative alleles of *qPL3* and *qTN4* showed significantly higher AGB than the varieties with the reference alleles. Our results suggest that *OsGA20ox1* and *NAL1* are promising genes for improving DW resistance in rice.

**Supplementary Information:**

The online version contains supplementary material available at 10.1186/s12284-023-00671-y.

## Background

While the rapid modernization of agriculture over the past half-century has led to increased food supply, it has also negatively impacted natural ecosystems (Tilman et al. 2011). One-quarter of global greenhouse gas emissions is estimated to come from agricultural activities including cultivation, fertilizer production, and land conversion of natural landscapes for crop production (Burney et al. 2010). The increased use of chemical fertilizers can cause negative impacts such as biodiversity loss, pollution, and eutrophication of water bodies (Mader et al. 2002; Dale and Polasky 2007; Wittwer et al. 2021). Excess application of herbicides and pesticides disturb natural ecosystems and increase the emergence of resistant weeds and pests (Islam et al. 2018; Sharma et al. 2019). Reducing the use of agrochemicals is expected to mitigate the negative impacts, though it could greatly reduce the crop yield and increase the risk of food shortage (Wittwer et al. 2021). Going forward, novel technologies for maintaining food production while minimizing the ecological impact are highly required for sustainable agriculture (Wittwer et al. 2021).

Reducing the use of herbicides in paddy fields can enable weed species such as *Echinochloa oryzicola*, *Monochoria vagnalis*, and *Cyperaceae microiria* to develop, all of which overwhelm rice paddies (Chauhan 2020). Hand weeding is the most reliable way to avoid loss of rice yield, but it is not practical for farmers who own several hectares of farmland. Instead, physical, chemical and biological approaches have been proposed for controlling paddy weeds. Weeding machines, which have blades and rotors, can remove weeds that have emerged from the soil between rows of rice (Pullen and Cowell 1997). The application of rice bran, the by-product of rice polishing, increases the electrical conductivity of the soil solution and suppresses the germination of *M. vaginalis* (Nozoe et al. 2012). Releasing Aigamo flightless ducks to the paddy field is another alternative method of removing harmful weeds and insects without human intervention (Tojo et al. 2007). These approaches are effective for sustaining the rice yield but are not without disadvantages: using a weeding machine is costly, applying large amounts of rice bran requires substantial labor, and ducks are often attacked by wild animals. Labor-saving and low-cost approaches for effective weed control are required for agriculture with reduced herbicide use.

Deep-water (DW) management is a possible option for effective weed control because excess water limits light penetration and gas supply to the weeds, resulting in their death (Kende et al. 1998). For instance, submerging the field in 15-cm deep water for 30 days suppresses more than 90% of survival in *E. oryzicola* and *C. microiria* (Arai and Miyahara 1956). DW management with a water depth of 10 cm for 19 days and subsequently 15 cm for 60 days has been reported to kill 99% of *E. oryzicola* (Tachibana 2014). Such management is especially effective at the early growth stage of rice, because these weeds are small and susceptible to growth inhibition (Tachibana 2014). DW management requires neither specialized equipment nor extensive labor, and can be easily applied in areas where there is sufficient irrigation water. However, the remaining limitation of this approach is that rice growth is also suppressed by hypoxia, especially due to the suppression of tiller buds’ emergence (Ohe et al. 1994, 2010; Watanabe et al. 2006; Ismail et al. 2012). Enhancing DW resistance to rice plants is essential to avoid suppressing their growth.

The advantages of DW management have long been recognized for several centuries by Japanese farmers (Nakamura and Hoshino 1944; Arai and Miyahara 1956; Ismail et al. 2012; He et al. 2023). They have grown large-size mature seedlings approximately 18 cm to 24 cm in height in upland rice nurseries and transplanted then to paddy fields by hand (Matsuo 1957). The mature seedlings are resistant to DW management because they have well-developed tillers and root systems, and a large part of the plant body is above the water surface (Matsuo 1957). The farming system significantly changed in 1960s when transplanting machines were introduced. Farmers began using nursery boxes to save space and improve efficiency for machine transplanting. The seedlings grown in the nursery boxes ranged from 7 to 13 cm height, which are inadequate for DW management (Matsuo 1957). With the widespread use of herbicides, however, DW management itself is no longer required by modern farmers. To revive DW management in modern agriculture instead of using herbicides, new rice varieties having DW resistance should be developed such that the immature seedlings grown in the nursery boxes can be used for transplanting.

DW resistance of rice species has been intensively studied in deepwater rice varieties. Deepwater rice varieties, which are cultivated in flooded area of South and Southeast Asia, enable rapid elongation of their internode more than 2 m by responding to increase of water level (Kende et al. 1998). It has been elucidated that *SD1* haplotype of deepwater rice leads to accumulation of active gibberellin species under submerged condition and generates remarkable internode elongation in the presence of *SNOKEL1/2* genes (Hattori et al. 2009; Kuroha et al. 2018). While the rice plants carrying these genes which are responsible for internode elongation of deepwater rice are potentially effective under DW managements for efficient weed control, there are risks of lodging and yield loss because internode elongation initiates in response to change of water depth even at the initial growth stages (Hattori et al. 2011). There have been few genetic studies of DW resistance specific to the conditions of moderate DW management for weed control.

The properties of varieties required for rice cultivation have also changed in the past decade. Landrace varieties, which have long been used by traditional farmers, usually have greater plant length with smaller tiller numbers, while the commercial varieties crossed by modern breeders have lower plant length with higher tiller numbers (Yang et al. 2006). Although the commercial varieties have high yield potential in modern rice cultivations, they may not always demonstrate their potential under DW conditions. The genetic diversities of growth and biomass production under DW conditions need to be clarified using diverse varieties including both landraces and commercial varieties.

In this study, we evaluate the natural genetic variation of DW resistance of 165 temperate *japonica* rice varieties. Here we defined the DW resistance as having high above-ground biomass (AGB) at the end of the DW treatment. We also analyze the association between AGB and plant length (PL) or tiller number (TN) as the key morphological traits associated with DW resistance. Finally, we conduct a genome-wide association study (GWAS) to identify genomic regions for PL and TN for the future development of DW resistant rice.

## Materials and Methods

### Plant Materials and Cultivation

We used 87 landrace rice varieties which were historically used by farmers (hereafter simply referred to as landraces), 76 commercial varieties which were bred by official breeding sectors (referred to as commercials), and two varieties whose histories are unknown, which were collected from various regions in Japan except Hokkaido (Yano et al. 2016; Additional file 2: Table S1). The rice seeds were sown in nursery boxes on 7 May 2020, and 6 May 2021. Seedlings at the fourth leaf stage were transplanted to paddy fields (alluvial clay loam) at the Tokyo University of Agriculture and Technology (35° 39′ N, 139° 28′ E) at 22.2 hills m^−2^ (15 cm × 30 cm) with one seedling per hill on 21 May 2020, and 19 May 2021. All plant residues from the previous year were chopped up and mixed into the soil during the winter, and N, P_2_O_5_, and K_2_O were applied at 3, 6, and 6 kg/10 a, respectively. One-third of the total nitrogen was applied as nitrogen sulphate; one-third as LP-50 elution-controlled urea (JCAM Agri Co., Ltd, Tokyo, Japan), and one-third as LPS-100 elution-controlled urea (JCAM Agri Co., Ltd, Tokyo, Japan). Topdressing was not applied.

We administered two water treatments, shallow-water (SW) and deep-water (DW), in the two adjacent paddy fields. The water depth was maintained at 5 cm (3.0–7.0 cm) in SW throughout the growth period. The water depth of DW was maintained at 20 cm (18.5–23.0 cm) for 1 to 4 weeks after transplanting and thereafter maintained at 5 cm. The actual duration of DW treatment was from 30 May (0 days after treatment; 0 DAT) to 26 June (+ 27 DAT) in 2020, and from 28 May (0 DAT) to 22 June (+ 25 DAT) in 2021. Each variety was grown in 2 rows of eleven hills in SW and in 2 rows of six hills in DW. All varieties were used as biological replicates. To exclude effects other than the differences in water depth, weed and insect controls were carried out using chemical pesticides and herbicides following conventional cultivation practices.

### Phenotyping

Plant length (PL) was repeatedly measured for SW and DW at − 1, + 3, + 10, + 17 and + 38 DAT in 2020, and at − 2, + 3, + 10, + 17, + 24 and + 31 DAT in 2021. The tiller number (TN) was counted for SW and DW at + 34 DAT in 2020 and at + 17 and + 33 DAT in 2021. Four hills in each variety were used for the measurements.

The above-ground biomass (AGB) was examined for DW at + 33 DAT in 2021. Eight hills of each variety were harvested at the soil surface. All samples were dried in a ventilated oven at 80 °C for at least 72 h and weighed. Because one of the commercial varieties ‘Ginbozu-midashi’ died in DW, the data was excluded from the analysis.

### Genome Wide Association Study (GWAS)

The 165 varieties were genotyped as described in previous studies (Yano et al. 2016; Nomura et al. 2021). We identified a total of 138,411 SNPs and InDels after removing nucleotide variations with missing rates of > 0.10 and a minor allele frequency of < 0.05. The genetic structure of the 165 varieties was analyzed using the “nipals” parameter of the package ‘pcaMethods’ (version 1.78.0) for R software (version 4.3.1). The GWAS was performed with the linear mixed model according to previous studies (Yano et al. 2016; Nomura et al. 2021):

1 $$ y = X\beta + Z\mu + \varepsilon $$

where *y* is a vector of phenotypes, *X* is the matrix of DNA polymorphisms, *β* is the vector of assumed fixed effects caused by DNA polymorphism, *Z* is the incidence matrix between *y* and *μ*, and *μ* is the random effects caused by the genetic background. The value of *μ* was assumed using N (0, *Kσ*^*2*^_*G*_), where *K* is the kinship matrix, and *σ*^*2*^_*G*_ is the genetic variance.* ε* is the matrix of residual effects and was assumed using N (0, *Iσ*^*2*^_*E*_), where *I* is an identity matrix, and *σ*^*2*^_*E*_ is the residual variance. The principal components (PC1 and PC2) as fixed effects were included in GWAS as based on the structure analysis described in Nomura et al. (2021). The resulting additive relation matrix was computed using the function “A.mat,” and the GWAS was performed using the “GWAS” function in the package “rrBLUP” (Endelman 2011) for R software with the modifications described in Yano et al. (2016). Additionally, we conducted Joint-GWAS (Müller et al. 2019). To combine all PL or TN data obtained for two years throughout the growth stage, we calculated Best Linear Unbiased Predictors (BLUPs) using the following model:

$$ {\text{y}} = \mu + {\text{G}} + {\text{E}} + \varepsilon $$

where y is phenotypic value, *μ* is grand mean value, G is the effect of genotype, E is all data points across two years, and *ε* is residual errors. The regression was conducted using the “H2cal” function in the “inti” package (Lozano-Isla 2023) for R software. In this GWAS, the threshold was set to – log_10_ (*p*) > 5. A LD map was drawn using the “LDheatmap” R package (Shin et al. 2006) with the sequence variant data from the 165 varieties. The LD block was used to determine candidate regions. A set of candidate gene IDs and descriptions was downloaded from the MSU Rice Genome Annotation Project and the Rice Annotation Project Database (Kawahara et al. 2013; Sakai et al. 2013).

### Sanger Sequencing

Genomic DNA from young plant leaves of four varieties (listed in Additional file 2: Table S2) were extracted using the cetyltrimethylammonium bromide (CTAB) method. The DNA concentration of each sample was measured using a Qubit fluorometer (Thermo Fisher Scientific, Hampton, NH, USA) and adjusted to 10 ng/μL. PCR primers were set to amplify and sequence around the 5.7 kb region of *OsGA20ox1* (from 2 kb above the start codon to 1.5 kb below the stop codon; 36,147,000–36,152,700 kb). Primers were designed on the basis of the IRGSP v1.0 *Oryza sativa* Nipponbare reference genome sequence listed in Additional file 2: Table S3. The PCR products were purified by a purification kit using the Exo-CIP Rapid PCR Cleanup Kit (New England Biolabs Japan, Tokyo, Japan). After verifying the presence of a single PCR product by electrophoresis using a 1% agarose gel, Sanger sequencing was performed at FASMAC Co., Ltd. (Kanagawa, Japan). The sequence data including the raw chromatograms was analyzed using the software, Snap-Gene Viewer 6.2 (USA).

### RNA Extraction and Quantitative Reverse-Transcription (qRT)-PCR

Seeds were sown in plastic pots filled with soil and then grown in a growth chamber under 14 h light irradiation at 400 μmol photon m^−2^ s^−1^ at 28/25 °C. The varieties used in this study are listed in Additional file 2: Table S2. The plants at two- to three- leaf stages were moved to a transparent plastic container (60 cm × 36 cm, 30 cm height) and submerged in water approximately 20 cm deep from the soil surface. The section from 0 to 1 cm above the basal part of the node, including meristems, was harvested 3 h after the DW treatment.

Frozen leaf samples were grinded using the TissueLyser (QIAGEN, Tokyo, Japan), and the total RNA was extracted with a Maxwell 16 LEV Plant RNA kit in a Maxwell 16 automated purification system (both Promega, Madison, WI, USA). RNA concentrations were measured with a Qubit fluorometer (Thermo Fisher Scientific, Hampton, NH, USA). Two micrograms of total RNA were converted to cDNA with the PrimeScript RT reagent Kit (TaKaRa, Japan). To quantify the expression levels of transcripts, qRT-PCR analysis was performed using the TB Green® Premix Ex Taq™ II (Tli RnaseH Plus) (TaKaRa, Japan) on a Step-One Plus Real-Time PCR system (Thermo Scientific, USA). The expression levels of target genes were normalized to the endogenous ubiquitin transcript level, *OsUBC32* (*Os02g0634800*). The comparative cycle threshold (ΔΔCt) method was used to calculate the relative expression levels of the target genes. The primer sequences of *OsGA20ox1*, *NAL1* and *UBQ2* used in this study were provided from Abe et al. (2012), Takai et al. (2013), and Ookawa et al. (2010) as listed in Additional file 2: Table S3.

### Statistical Analysis

All statistical analyses were performed in R version 4.3.1 software (R Core Team 2023). A two-tailed Welch’s t-test was performed using the “t-test” function of R software to compare the phenotypic trait values between the groups of landraces and commercials and between varieties with the reference and alternative. Correlation coefficients between traits were calculated using the “cor” function of R software. A Tukey-Kremer HSD test was performed using the “TukeyHSD” function of R software to compare the relative expression between varieties with the reference and alternative. A Steel–Dwass test was performed using the “pSDCFlig” function with the “Asymptotic” method of the package “NSM3” (version 1.16) of R software to compare the multiple groups classified by genotype.

## Results

### Genetic Variations in PL and TN Among Temperate *Japonica* Varieties

**Fig. 1:**
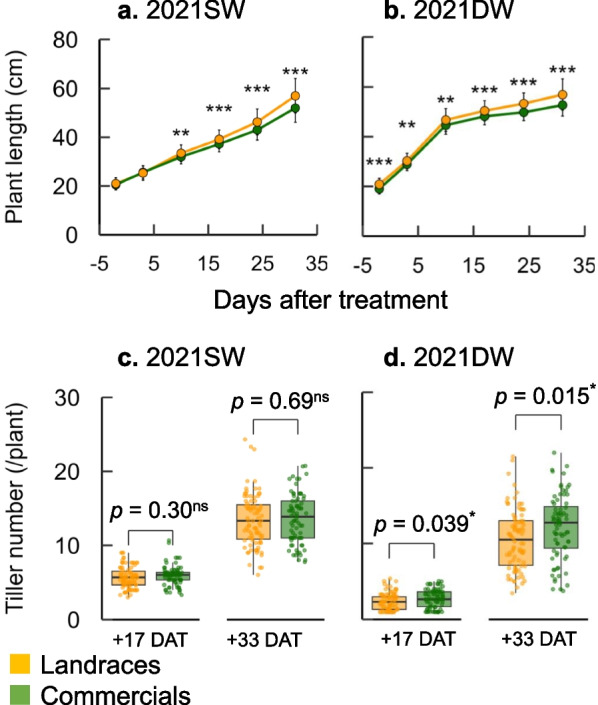
Plant length (PL) and tiller number (TN) during water treatments in 2021.** a**, **b** Changes in PL under shallow-water (SW) and deep-water (DW) of 165 temperate *japonica* varieties. **c**, **d** Changes in TN under SW and DW of 165 temperate *japonica* varieties. Differences between landraces (yellow) and commercials (green) were analyzed by Welch’s t-test (*p < 0.05, **p < 0.01, ***p < 0.001)

**Fig. 2:**
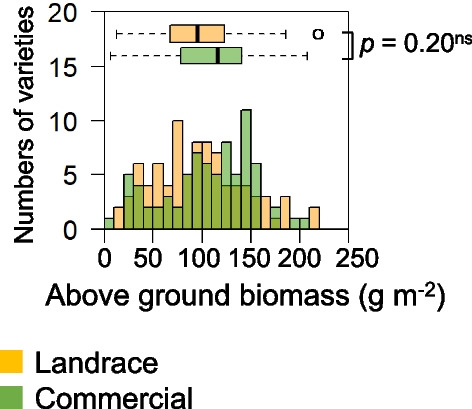
Histogram of above-ground biomass (AGB) under deep-water (DW) of 165 temperate *japonica* varieties in 2021. The rice plants were harvested at 33 days after treatment. Statistical difference between landraces (yellow) and commercials (green) was analyzed by Welch’s t-test. A dead variety during the water treatment was removed from the analysis

We compared the growth responses to SW (5 cm depth of water) and DW (20 cm depth of water) among 165 temperate *japonica* varieties (Fig. 1; Additional file 1: Fig. S1). Under SW in 2021, PL gradually increased from − 2 DAT to + 33 DAT (Fig. 1a). Under DW in 2021, PL rapidly increased in the initial growth phase (from − 2 DAT to + 10 DAT) and the growth slowed after + 10 DAT (Fig. 1b). The TN was greatly reduced at + 17 DAT under DW relative to SW, while the difference diminished at + 33 DAT (Fig. 1c, d). We next focused on the difference between landraces and commercials. Landraces had significantly greater PL than commercials under both SW and DW across all samplings except for the first two samplings under SW in 2021. In contrast, landraces had significantly smaller TNs than commercials at both + 17 and + 33 DAT under DW, and similar tendency was found under SW in 2021. In the 2020 experiment, we also found that landraces had greater PL and smaller TN than commercials under both SW and DW (Additional file 1: Fig. S1). The ranges of AGB at + 33 DAT under DW were 13.6–219.4 g m^−2^ in landraces and 6.2–207.4 g m^−2^ in commercials (Fig. 2). The mean values of AGB were similar between landraces and commercials.

**Fig. 3:**
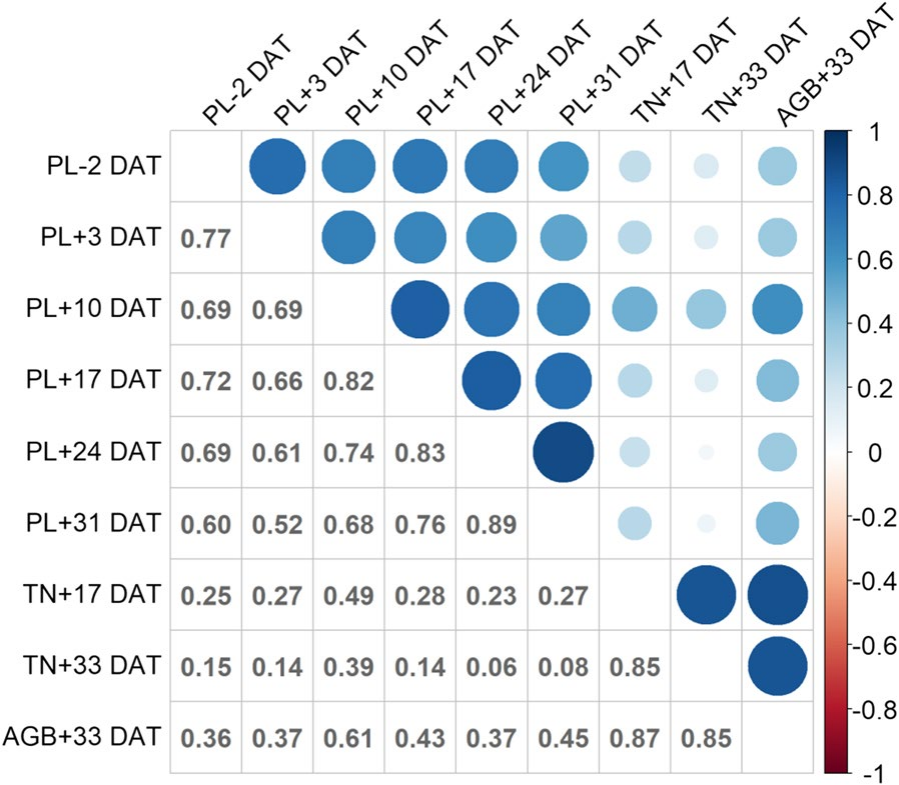
Pearson’s correlation matrix for traits under deep-water (DW) in 2021. The traits analyzed are plant length (PL), tiller number (TN), and above-ground biomass (AGB). Numbers on the lower triangular matrix indicate correlation between traits. The circle size indicates the strength of correlation and blue indicates a positive correlation (r = 1). DAT: days after treatment

We examined Pearson’s correlation coefficients between the traits under DW in 2021 (Fig. 3; correlations across the experimental years are shown in Additional file 1: Fig. S2). In 2021, all PL values at different growth stages positively correlated with AGB at + 33 DAT (Fig. 3). The highest correlation coefficient was found between PL at + 10 DAT and AGB at + 33 DAT (r = 0.61), indicating that the increased plant elongation in the earlier period contributes to enhanced biomass production under DW. We also found that the TN at both + 17 DAT and + 33 DAT highly correlated with AGB at + 33 DAT (Fig. 3).

### Identification of Genomic Regions Associating with PL and TN

**Fig. 4:**
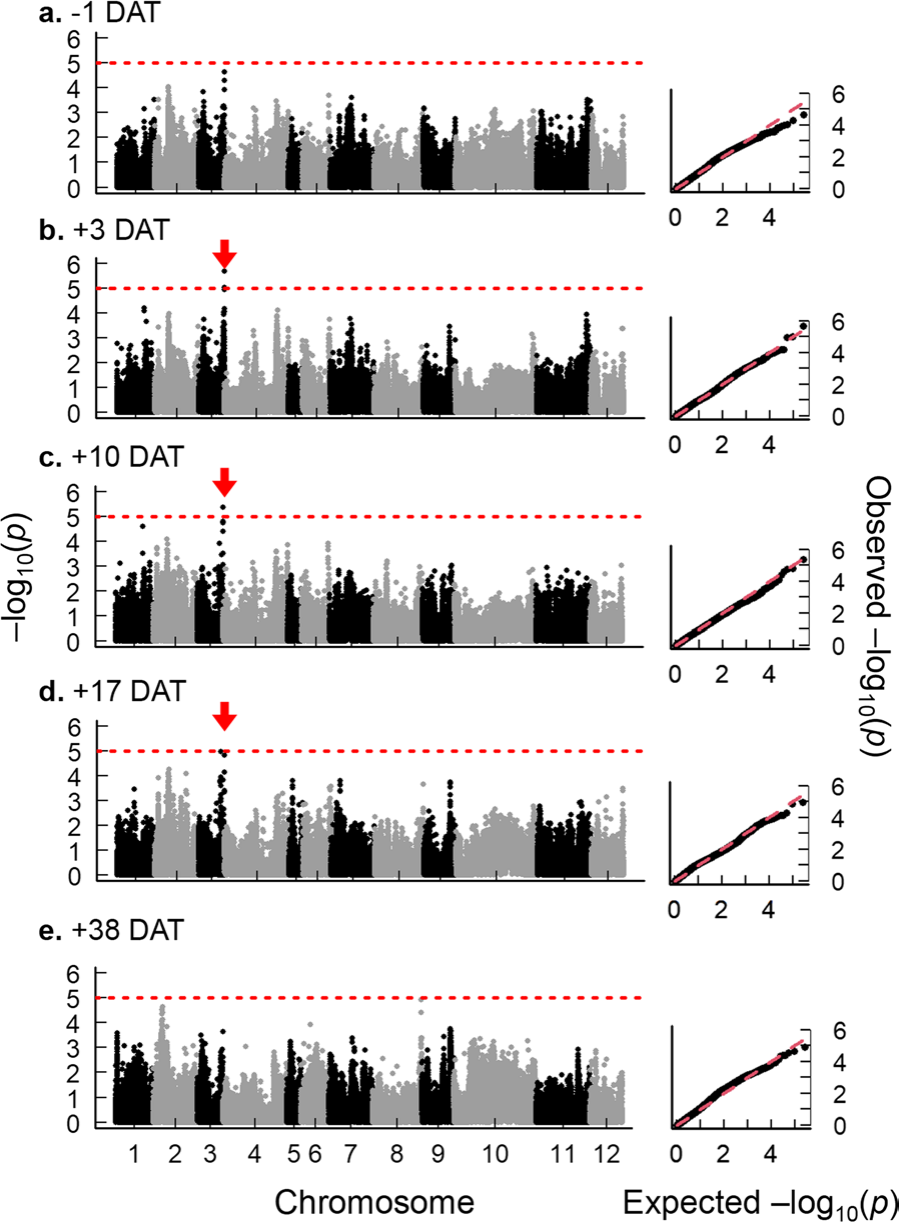
Manhattan plots and quantile–quantile (Q-Q) plots of plant length (PL) under deep-water (DW) in 2020. (**a**) − 1 days after treatment (DAT): (**b**) + 3 DAT: (**c**) + 10 DAT: (**d**) + 17 DAT: (**e**) + 38 DAT. The x-axis indicates the SNPs or indels physically mapped on each chromosome. The red arrows indicate the candidate region. The red dashed lines indicate the threshold lines (− log_10_ (*p*) = 5) set in this study

To identify the genetic regions associated with DW resistance traits, we conducted a GWAS for PL and TN. For PL in DW, a strong peak above the threshold (− log_10_(*p*) > 5) was identified on chromosome 3L at + 3, + 10 and + 17 DAT in 2020 and + 24 DAT in 2021 (Fig. 4; Additional file 1: Fig. S3; Table 1). The candidate region of this QTL (*qPL3*) was positioned between 35.0 and 36.3 Mb. Within the linkage disequilibrium block of this peak, *OsGA20ox1*, a gene encoding gibberellin oxidase was observed (Table 1; Additional file 2: Table S4; Additional file 1: Fig. S8a; Abe et al. 2012; Yano et al. 2012). Varieties with an alternative allele in this region showed significantly higher PL at + 10 DAT than those with the reference (Nipponbare) allele (Additional file 1: Fig. S8b). We also found that the alternative allele of *qPL3* exists more frequently in landraces (79%) than in commercials (21%) (Additional file 1: Fig. S8c), which is consistent with the greater PL in landraces than in commercials under DW. The other peaks for PL were also detected on chromosome 5S at + 24 DAT in 2021 and on chromosome 12L at + 3 DAT in 2021 (Additional file 1: Fig. S3) in DW. In SW, peaks were identified on chromosome 2S at + 3 DAT in 2020 and + 10 and + 24 DAT in 2021, and on chromosome 12L at + 3, + 10 and + 17 DAT in 2021 (Additional file 1: Figs. S4; S5). To confirm existences of these QTLs, we conducted a joint analysis of GWAS using the extracted genetic effects from all datapoints of PL by BLUPs (see details in Materials and Methods). Under DW, *qPL3* was also detected in this analysis (Additional file 1: Fig. S7b). Under SW, we found a peak on chromosome 2S similar position of the separate analysis (Additional file 1: Figs. S4; S5; S7a). We also found a peak on chromosome 12L under both DW and SW (Additional file 1: Fig. S7a,b).

**Table 1:** Summary information of quantitative trait locus (QTLs) for the traits related with deep-water resistance detected by genome-wide association study

QTL	Trait	Year	Chromosome	Start (Mbp)	End (Mbp)	−Log_10_(*p*)
*qPL2*	PLSW + 3 DAT PLSW + 10,24 DAT	2020 2021	2	6.0	14.0	5.41
*qPL3*	PLDW + 3,10,17 DAT PLDW + 24 DAT	2020 2021	3	32.5	36.5	5.70
*qPL5*	PLDW + 24 DAT	2021	5	4.0	8.0	5.52
*qPL12*	PLDW + 3 DAT PLSW + 3,10,17 DAT	2021 2020	12	22.0	Terminus	5.70
*qTN1*	TNSW + 17 DAT	2021	1	11.0	25.0	5.33
*qTN4*	TNDW + 34 DAT TNDW + 33 DAT TNSW + 34 DAT	2020 2021 2020	4	28.0	34.0	8.62
*qTN5*	TNSW + 33 DAT	2021	5	25.0	Terminus	5.84
*qTN10*	TNDW + 34 DAT	2020	10	9.0	13.0	5.38

PL: plant length, TN: tiller number, DAT: days after treatment, SW: shallow-water, DW: deep-water

**Fig. 5:**
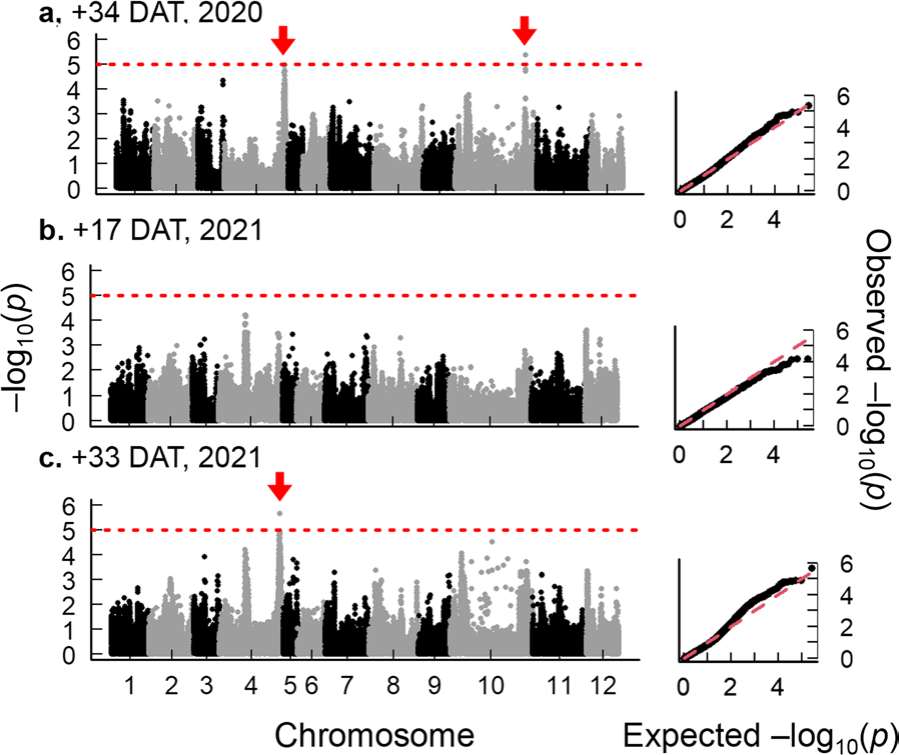
Manhattan plots and quantile–quantile (Q-Q) plots of tiller number (TN) under deep-water (DW). (**a**) + 34 days after treatment (DAT) in 2020: (**b**) + 17 DAT in 2021: (**c**) + 33 DAT in 2021. The x-axis indicates the SNPs or indels physically mapped on each chromosome. The red arrows indicate the candidate region. The red dashed lines indicate the threshold lines (− log_10_ (*p*) = 5) set in this study

For TN in DW, a strong peak was detected on chromosome 4L at + 34 DAT in 2020 and + 33 DAT in 2021 (Fig. 5). The candidate region of this QTL (*qTN4*) positioned from 28.0 to 34.0 Mb. *LOC_Os04g52479(Os04g0615000),* or *Narrow Leaf 1 (NAL1)* was observed within the linkage disequilibrium block of this peak (Table 1; Additional file 2: Table S4; Additional file 1: Fig. S8d). Varieties with alternative allele in this region showed significantly higher TN at + 33 DAT than those with reference (Nipponbare) allele (Additional file 1: Fig. S8e). The proportion of the alternative allele was 42.5% in landraces and 57.5% in commercials (Additional file 1: Fig. S8f). Another peak was also found on chromosome 10S at + 34 DAT under DW in 2020 (Fig. 5). Under SW, a strong peak was also identified on chromosome 4L at + 34 DAT in 2020 at the same loci of *qTN4* (Additional file 1: Fig. S6). The other peaks were identified on chromosome 1S at + 17 DAT and on chromosome 5L at + 33 DAT under SW in 2021 (Additional file 1: Fig. S6). From the joint analysis of GWAS, *qTN4* was also detected for TN under both SW and DW (Additional file 1: Fig. S7c,d).

### Sequence and Expression Analysis of Candidate Genes of *qPL3* and *qTN4*

**Fig. 6:**
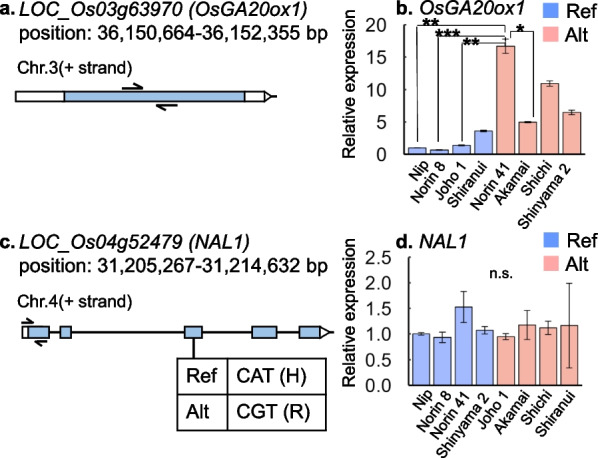
Detailed analysis of candidate genes. **a,c**: The DNA mutations in the coding region of (**a**) *OsGA20ox1* and (**c**) *NAL1.* “Ref” and “Alt” indicate the reference and alternative genotype, respectively. The black arrows in (**a**) and (**c**) indicates the primer positions for qRT-PCR analysis. **b,d**: The expression patterns of (**b**) *OsGA20ox1* and (**d**) *NAL1* between varieties with reference and alternative genotypes in basal nodes under 3 h after deep-water treatment determined by qRT-PCR analysis. The relative expression in Nipponbare (Nip) was set to 1. The *OsUBC32* gene was used as an internal control. Data are represented as mean ± SD, n = 4 biologically independent samples. Differences between the varieties with reference genotype and alternative genotype were analyzed by Tukey-Kremer HSD test (*p < 0.05, **p < 0.01, ***p < 0.001). Akamai: Akamai_Nagasaki, Joho 1: Johoibaraki 1, Shichi: Shichimenchomochi, Shinyama 2: Shinyamabuki 2

Although there were SNPs at 107,185 bp upstream of the start codon and at 126,212 bp downstream of the terminus codon of *OsGA20ox1*, no genomic mutation was found in the *OsGA20ox1* locus in our next generation sequence data. We then analyzed the 5.7 kb region of *OsGA20ox1* (from approximately 2 kb above the start codon to 1.5 kb below the stop codon; 36,147,000–36,152,700 kb) of the varieties “Norin 41,” “Shichimenchomochi,” “Johoibaraki 1,” and “Norin 8” by Sanger sequencing (Additional file 2: Table S2; Additional file 1: Fig. S9). The analysis showed that neither the coding region nor peripheral region of *OsGA20ox1* had any genome mutation among the varieties (Additional file 1: Fig. S9). For qRT-PCR analysis, we took samples of the basal part of the leaves from plants 3 h after DW treatment. The relative expression of *OsGA20ox1* was higher in the varieties with the alternative genotype of *qPL3* than those with the reference genotype (Fig. 6b).

There was a SNP with a high -log_10_ (*p*) value for TN in the *NAL1* region. The SNP was located on the third exon of *NAL1*, and the encoded amino acid was substituted from histidine (CAT) to arginine (CGT) (Fig. 6c). There was less difference in the relative expression of *NAL1* between the varieties with the reference and alternative genotype when using samples of the basal part of leaves from plants 3 h after DW treatment (Fig. 6d).

### Combined Effect of *qPL3* and *qTN4* for Improving DW Resistance

**Fig. 7:**
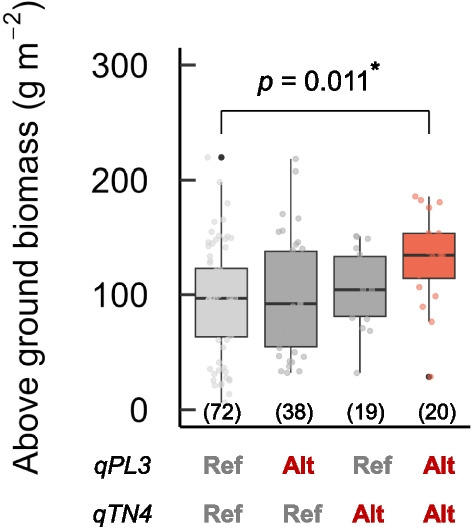
Combined effects of *qPL3* and *qTN4* under deep-water (DW). Box plots of the above-ground biomass (AGB) by the genotypes classified at the peak marker position. “Ref” and “Alt” indicate the reference and alternative genotypes, respectively (based on the Nipponbare genome). The number of parentheses indicates the number of varieties. Six varieties missing either the *qPL3* or *qTN4* genotype were removed from the analysis. Differences between the genotypes were analyzed by Steel–Dwass test (*p* < 0.05)

We examined the effects of the genotypes of *qPL3* and *qTN4* on AGB after the DW treatment (Fig. 7). Although the varieties having one alternative genotype at either *qPL3* or *qTN4* showed similar AGB to those having the reference genotypes at both QTLs, the varieties having alternative genotypes at both QTLs showed significantly higher AGB than those having the reference genotypes at both QTLs, indicating that these QTLs are effective for improving DW resistance in rice plants at the initial growth stage after transplanting.

## Discussion

DW management is highly effective for suppressing weed growth in organic rice farming (Sasaki et al. 1994), but it simultaneously reduces the rice growth rate and, subsequently, the yield (Ohe et al. 1994; Watanabe et al. 2006). Identifying genomic factors that enhance rice growth at the initial growth stage under DW conditions will contribute to improving breeding programs. The molecular mechanisms of the internode elongation of deepwater rice varieties under an extreme flooding have been well studied (Hattori et al. 2009; Kuroha et al. 2018; Nagai et al. 2020). In contrast, few genetic studies have been conducted on DW resistance under partially submerged conditions for weed suppression. In this study, we investigated the genetic differences in PL and TN, which may be associated with DW resistance, among the temperate *japonica* rice panel and performed a GWAS to identify causal genes.

### PL and TN Closely Relate to DW Resistance

We found a large difference in AGB at + 33 DAT within the range of 0 to ~ 200 g m^−2^ among the 165 template *japonica* varieties, indicating large genetic variation in DW resistance (Fig. 2). The AGB closely correlated with PL at + 10 DAT, TN at + 17 DAT, and TN at + 33 DAT, supporting our hypothesis that PL and TN are linked to DW resistance (Fig. 3). It is known that greater PL facilitates the transportation of oxygen from the upper leaves to the submerged organs, keeping the respiration rate for maintaining homeostasis (Voesenek and Bailey-Serres 2015). Our result suggests that the greater PL at the initial growth stage prevents the complete submergence of the plant and promotes growth until the end of DW treatment (Fig. 1). A higher TN indicates a greater photosynthetic leaf area above water, promotes the supply of O_2_ from the top leaves to the roots, and enhances the root distribution underground, which increases biomass production (Shimono and Okada 2013). Our results suggest that there is a large genetic variation in the activity of tiller bud production even under DW, leading to the difference in DW resistance (Fig. 1). It should be noted that the correlation between PL and TN was weak (Fig. 3; Additional file 1: Fig. S2), suggesting that the genomic factors regulating PL and TN differ under DW. The independent selection of PL and TN would be effective for improving DW resistant rice.

We found no intrinsic difference in AGB between landraces and commercials, although there were differences in PL and TN (Figs. 1, 2). This result was beyond expectations because we hypothesized that landraces would have higher DW resistance from being adapted to the traditional Japanese farming systems. As mentioned in the background, farmers used to grow large mature seedlings in upland nurseries before transplanting machines and nursery boxes were introduced. Our result suggests that the properties of seedlings grown in nursery boxes are different from those grown in upland nurseries, and some landraces grown in nursery boxes may not have DW resistance.

### Genomic Regions for Increasing PL under DW

We detected *qPL3* under DW at + 3, + 10, + 17 DAT in 2020 and at + 24 DAT in 2021 in our GWAS scan (Fig. 3; Additional file 1: Figs. S3; S7). The LD analysis shows that the genomic region of *qPL3* contains the gene encoding gibberellin biosynthesis *OsGA20ox1* (chr3: 36,150,664–36,152,355). GA20 oxidase has been known to catalyze two parallel pathways, GA_53_ to GA_20_ and GA_12_ to GA_9_, which are then converted into active gibberellin species, GA_1_ and GA_4_, respectively, by GA3 oxidase (Ashikari et al. 2002; Oikawa et al. 2004). Plants overexpressing *GA20ox* genes show rapid stem elongation as active GA concentrations increase in rice and *A. thaliana* (Huang et al. 1998; Coles et al. 1999; Oikawa et al. 2004). Rice lines with a high expression of *OsGA20ox1* showed high seedling vigor and shoot elongation (Abe et al. 2012). Furthermore, rice lines with a high expression of *OsGA20ox2 (sd1)*, a homolog of *OsGA20ox1*, under the completely submerged condition showed rapid internode elongation (Kuroha et al. 2018). The current study revealed that the varieties with an alternative genotype at *qPL3* had greater PL and relative expression of *OsGA20ox1* than those with the reference genotype (Fig. 6b; Additional file 1: Fig. S8), strongly suggesting that the *OsGA20ox1* gene is responsible for regulating the growth under DW at the initial growth stage underlying *qPL3*.

The Sanger sequencing analysis showed no base substitution in the genomic regions around *OsGA20ox1* among the four varieties with different genotypes (Fig. 6; Additional file 1: Fig. S9; Additional file 2: Table S2). Abe et al. (2012) found no nucleotide changes in the coding region between Dunghan Shali and Iwatekko which are the temperate *japonica* varieties having different expression levels of *OsGA20ox1*. They hypothesized that some of the four DNA changes within the 10-kb region upstream of the *OsGA20ox1* (6 bp insertion at − 9220 bp, a nucleotide substation at − 5609 bp, 10 bp insertion at − 2320 bp, and 1 bp deletion at − 532 bp of the translation start site) affected the expression changes, although this has not yet been validated. In the current study, we found no DNA change at − 2320 bp and − 532 bp upstream of *OsGA20ox1* among the four varieties, though we did not analyze the more upstream sequence. These unidentified mutations may have caused the observed difference in the expression levels in this study.

*qPL3* was repeatedly identified under DW, while no GWAS peak at the *qPL3* location was found under SW (Fig. 4; Additional file 1: Figs. S4; S5; S7). This suggests that the genetic effect of *qPL3* depends on the environments in which rice plants grow. Our follow-up validation showed that the varieties with the alternative genotype of *qPL3* had greater PL at + 10 DAT in 2020 than the varieties with the reference genotype in DW, whereas its difference was reduced under SW (Additional file 1: Fig. S10). Such unique responses may be attributed to hormonal interactions. It has been reported that submerged plants start accumulating ethylene, which in turn promotes GA biosynthesis (Kende et al. 1998; Kuroha et al. 2018). The alternative genotype of *OsGA20ox1,* which strongly interacts with ethylene signaling may produce higher levels of active GA species than the reference genotype under DW. The molecular network underlying *GA20ox1* responses to DW environments should be further investigated in future studies.

### Genomic Regions for Increasing TN under DW

*qTN4* was detected at + 34 DAT under both SW and DW in 2020 and + 33 DAT under DW in 2021 (Fig. 5; Additional file 1: Figs. S6; S7). We found that *LOC_Os04g52479(Os04g0615000),* known as *Narrow Leaf 1*(*NAL1*), is located in the region of *qTN4*. *NAL1* encodes a putative trypsin-like serine/cysteine protease and plays a key regulatory role in leaf morphological development by affecting polar auxin transport (Qi et al. 2008; Li et al. 2023). Yano et al. (2016) determined that *NAL1* controls panicle number by conducting a GWAS using the same rice panels as the current study and validating their findings using transgenic plants. Similar results of *NAL1* on panicle number have also been reported by Ouyang et al. (2022). In our study, we found that the alternative genotype increases TN at the initial growth stages relative to the reference genotype (Additional file 1: Fig. S8). Therefore, *NAL1* can be considered a candidate gene for regulating TN under both SW and DW. We determined the SNP on the third exon of *NAL1*, leading to an amino acid change from histidine (H) to arginine (R) (Fig. 6c), which is identified as the main reason for the variation in the phenotypes regulated by *NAL1* (Taguchi-Shiobara et al. 2015). This suggests that *NAL1* is the causal gene regulating the TN in *qTN4*.

### Maximization of Rice Productivity under Reduced Agrochemicals for Sustainable Agriculture

Our results suggest that utilizing two QTLs, *qPL3* and *qTN4* would enhance the DW resistance of rice. However, we must consider the side effects of introducing these genes. For example, taller plants are more likely to lodge at the mature stage (Chandler Jr., 1969). In addition, the mutated allele of *NAL1* decreases the spikelet number per panicle at the expense of increasing panicle number (Yano et al. 2016). These negative effects should be mitigated by genetic and cultivation methods to maximize the rice productivity.

In addition to PL and TN, other properties may correlate with the DW resistance of rice. The formation of root aerenchyma, which is promoted under submerged conditions, enhances gas transport from the leaves to the roots (Colmer 2003; Evans 2004). The formation of radial oxygen loss (ROL) barriers in roots under submerged conditions reduces the loss of oxygen into the rhizosphere (Colmer 2003; Nishiuchi et al. 2012). A microlayer of gas (gas films) on the surface of submerged leaves maintains respiration and photosynthesis under submergence due to the enlarged water–gas interface between the leaf and surrounding water (Pedersen et al. 2009; Kurokawa et al. 2018). Furthermore, some rice varieties of the *indica* subpopulation with *Submergence1 (Sub1)*, such as FR13A, show higher submergence resistance as a result of reduced energy consumption during respiration (Xu et al. 2006). The combination of these traits may further increase the DW resistance.

Varieties with high DW resistance may be useful for effective weed control with reduced chemical herbicide use, and also exhibit vigorous growth by accumulating other traits such as efficient nutrient use with reduced chemical fertilizer use in sustainable agriculture. Utilizing landraces as breeding material is a potential option for improving the target traits as shown in previous studies (Chigira et al., 2020; Nomura et al. 2021), although landraces also have undesirable traits such as low yield and susceptibility to lodging. Furthermore, the combination of new rice varieties and various methodologies for cultivation with reduced agrochemicals may be able to reduce the water level of DW management and promote rice growth under low nutrient conditions. The development of optimal cultivation methods including designing new rice varieties to maximize rice productivity with reduced or no agro-chemicals, remains a challenge for sustainable agriculture.

## Conclusion

DW management in rice fields is efficient for controlling paddy weeds with reduced herbicide use, though it also suppresses rice growth. In this study, we investigated a genetic variation of DW resistance among 165 temperate *japonica* varieties and conducted the GWAS for identifying the causal genes. The genetic variation of AGB under DW closely correlated with PL and TN at the early growth stage. The GWAS scan revealed that *qPL3* and *qTN4* were major QTLs for controlling PL and TN under DW, respectively. The *qPL3* region included *OsGA20ox1* encoded gibberellin biosynthesis. The varieties with an alternative genotype of *qPL3* have higher expression levels than those with the reference genotype. The *qTN4* region included *NAL1,* which correlates to leaf morphology and panicle number. A nucleotide mutation was found in the third exon of *NAL1,* possibly related to phenotypic change. The varieties with alternative alleles of both QTLs had larger AGB than those with the reference allele under DW. These results suggest that *OsGA20ox1* and *NAL1* are key genes for enhancing DW resistance in rice, although further experiments are needed to verify the physiological functions of these genes.

### Supplementary Information

**Additional file 1**.**Fig. S1**. Plant length (PL) and tiller number (TN) during the water treatments in 2020. **Fig. S2**. Pearson’s correlation matrix for the traits under deep water (DW) between 2020 and 2021. **Fig. S3**. Manhattan plots and quantile–quantile (Q−Q) plots of plant length (PL) under deep water (DW) in 2021. **Fig. S4**. Manhattan plots and quantile–quantile (Q–Q) plots of plant length (PL) under shallow water (SW) in 2020. **Fig. S5**. Manhattan plots and quantile–quantile (Q–Q) plots of plant length (PL) under shallow water (SW) in 2021. **Fig. S6**. Manhattan plots and quantile–quantile (Q–Q) plots of tiller number (TN) under shallow water (SW). **Fig. S7.** Manhattan plots and quantile–quantile (Q–Q) plots of plant length (PL) and tiller number (TN) analyzed by Joint–GWAS. **Fig. S8.** Detailed analysis of* qPL3* and *qTN4*. **Fig. S9.** Nucleotide and amino acid sequences of 5.7 kb region of *OsGA20ox1*. **Fig. S10.** Histogram of plant length (PL) at +10 days after treatment (DAT) in 2020.

**Additional file 2**.**Table S1**. Temperate *japonica* 165 varieties used in phonotyping and GWAS. **Table S2.** Varieties with different genotypes of *qPL3* and *qTN4* used for qRT-PCR and sequencing of candidate genes. **Table S3.** Primers used in this study. **Table S4.** List of candidate genes with amino acid replacements or deletions or mutations in promoter region. **Table S5.** Varieties belonging to four genotypes about *qPL3* and *qTN4*.

## Data Availability

The datasets supporting the conclusions of this article are included in this published article and its supplementary information files.

## Abbreviations

AGB: Above ground biomass production
DAT: Days after treatment
DW: Deep-water
GWAS: Genome-wide association study
PL: Plant length
QTL: Quantitative trait locus
SW: Shallow-water
TN: Tiller number
