# Supplementary material for: Identification of Genomic Regions for Deep-Water Resistance in Rice for Efficient Weed Control with Reduced Herbicide Use
Source: Rice (N Y). 2023 Nov 25;16:53. doi: 10.1186/s12284-023-00671-y (PMC10676340; doi:10.1186/s12284-023-00671-y)
Supplement: Supplementary file 1 — Additional file 1.Fig. S1. Plant length (PL) and tiller number (TN) during the water treatments in 2020. Fig. S2. Pearson’s correlation matrix for the traits under deep water (DW) between 2020 and 2021. Fig. S3. Manhattan plots and quantile–quantile (Q−Q) plots of plant length (PL) under deep water (DW) in 2021. Fig. S4. Manhattan plots and quantile–quantile (Q–Q) plots of plant length (PL) under shallow water (SW) in 2020. Fig. S5. Manhattan plots and quantile–quantile (Q–Q) plots of plant length (PL) under shallow water (SW) in 2021. Fig. S6. Manhattan plots and quantile–quantile (Q–Q) plots of tiller number (TN) under shallow water (SW). Fig. S7. Manhattan plots and quantile–quantile (Q–Q) plots of plant length (PL) and tiller number (TN) analyzed by Joint–GWAS. Fig. S8. Detailed analysis of qPL3 and qTN4. Fig. S9. Nucleotide and amino acid sequences of 5.7 kb region of OsGA20ox1. Fig. S10. Histogram of plant length (PL) at +10 days after treatment (DAT) in 2020. [file 12284_2023_671_MOESM1_ESM.pdf]

Additional file 1.

Supplementary Information for

Identification of genomic regions for deep-water resistance in rice for efficient weed control with reduced herbicide use

Marina Iwasa, Koki Chigira, Tomohiro Nomura, Shunsuke Adachi, Hidenori Asami, Tetsuya Nakamura, Takashi Motobayashi and Taiichiro Ookawa*

* Corresponding author: Taiichiro Ookawa, Graduate School of Agriculture, Tokyo University of Agriculture and Technology, 3-5-8 Saiwai-cho, Fuchu, Tokyo 183-8509, Japan; E-mail: ookawa@cc.tuat.ac.jp

This PDF file includes:
Figs. S1 to S10.

Tables S1 to S5 are included in the **Additional file 2**.

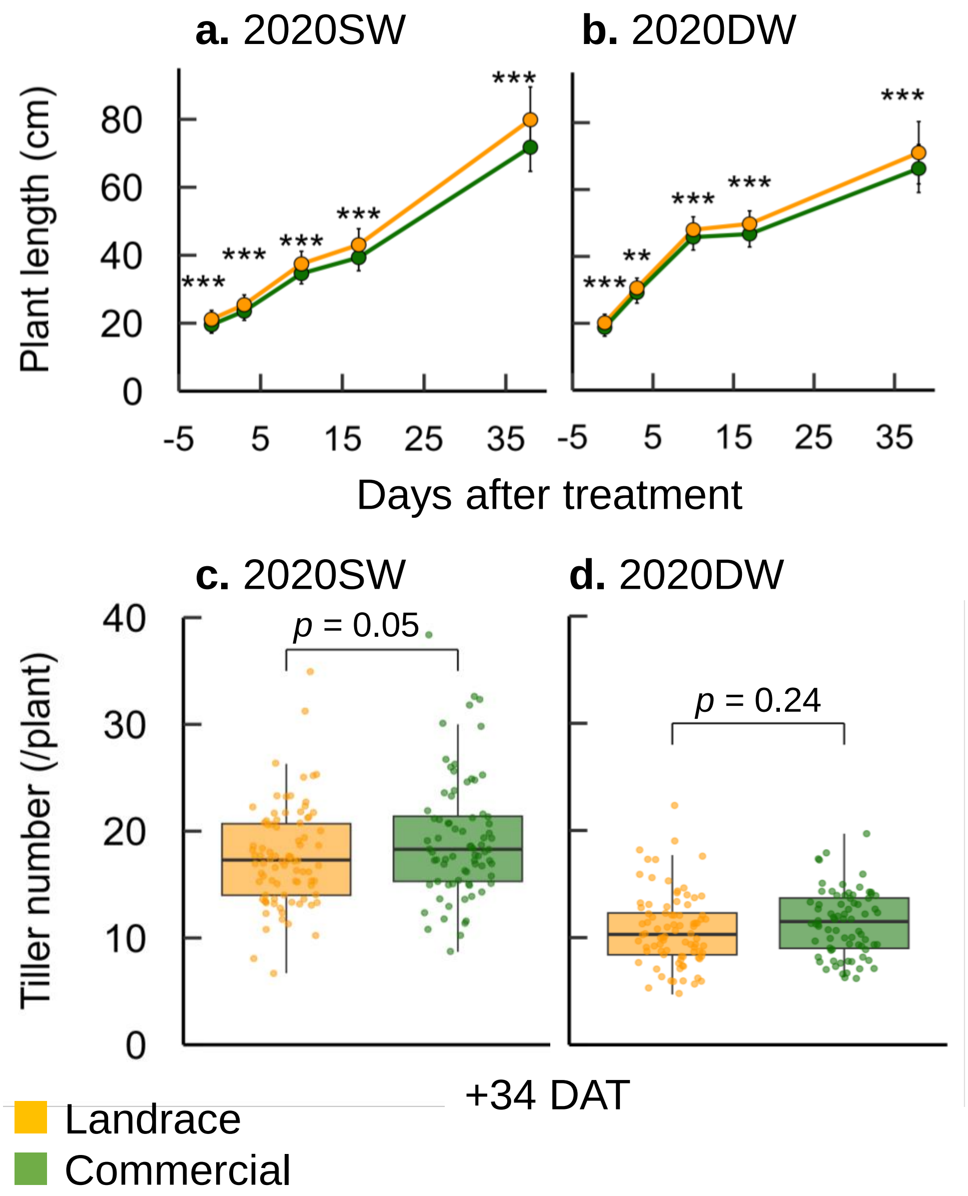

Figure S1.
Plant length (PL) and tiller number (TN) during the water treatments in 2020.

a,b: Changes of PL under shallow-water (SW) and deep-water (DW) of 165 temperate *japonica* varieties.

c,d: Changes of TN under SW and DW of 165 temperate *japonica* varieties.

Data are represented as mean ± SD (n = 3) of either landraces (yellow) or commercials (green). Differences between landraces and commercials were analyzed by Welch's t-test (*: $p < 0.05$, **: $p < 0.01$, ***: $p < 0.001$).

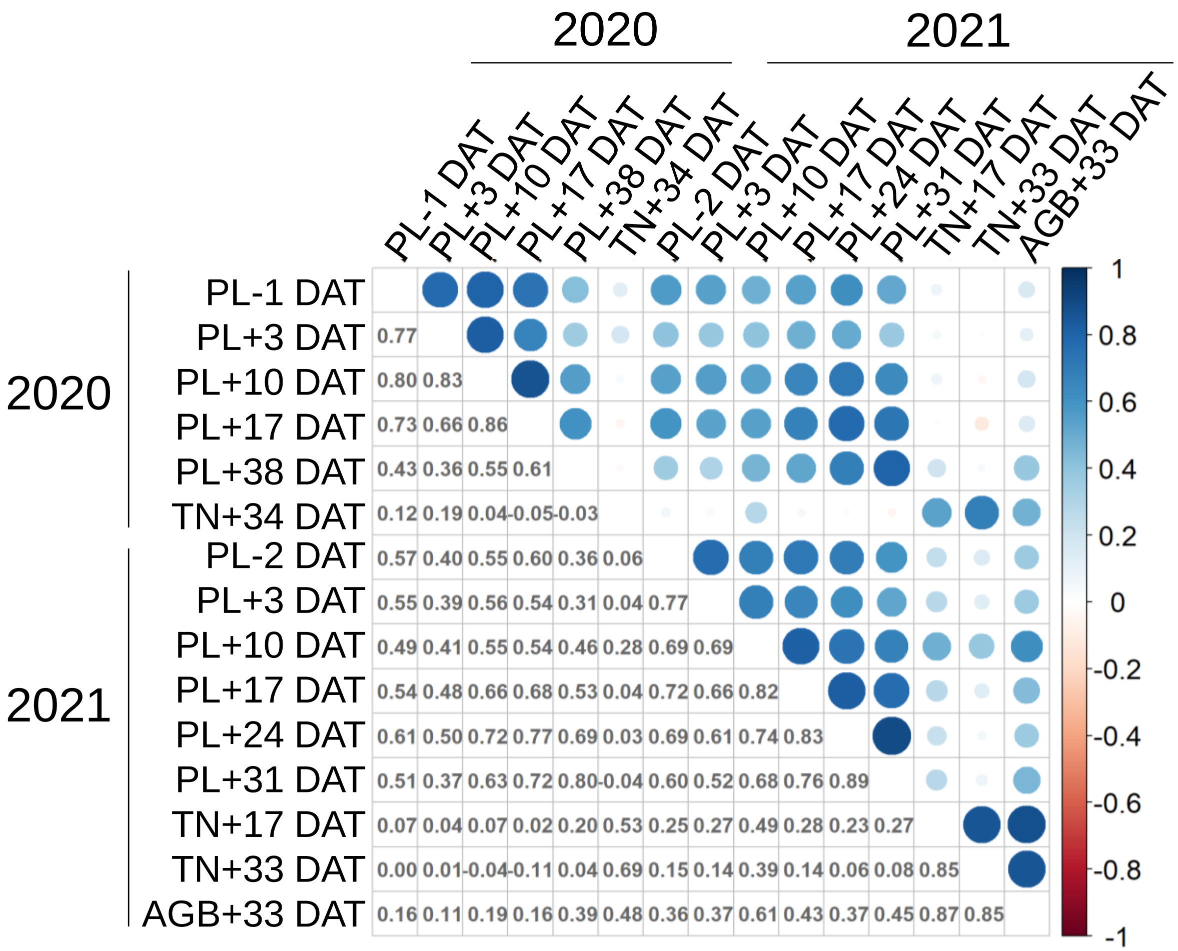

Figure S2.
Pearson's correlation matrix for the traits under deep-water (DW) between 2020 and 2021.
The traits that analyzed are plant length (PL), tiller number (TN), and above-ground biomass (AGB). Numbers on the lower triangular matrix indicate correlation between traits. The size of circle indicates the strength of correlation and blue indicates positive correlation (r = 1). DAT: days after treatment.

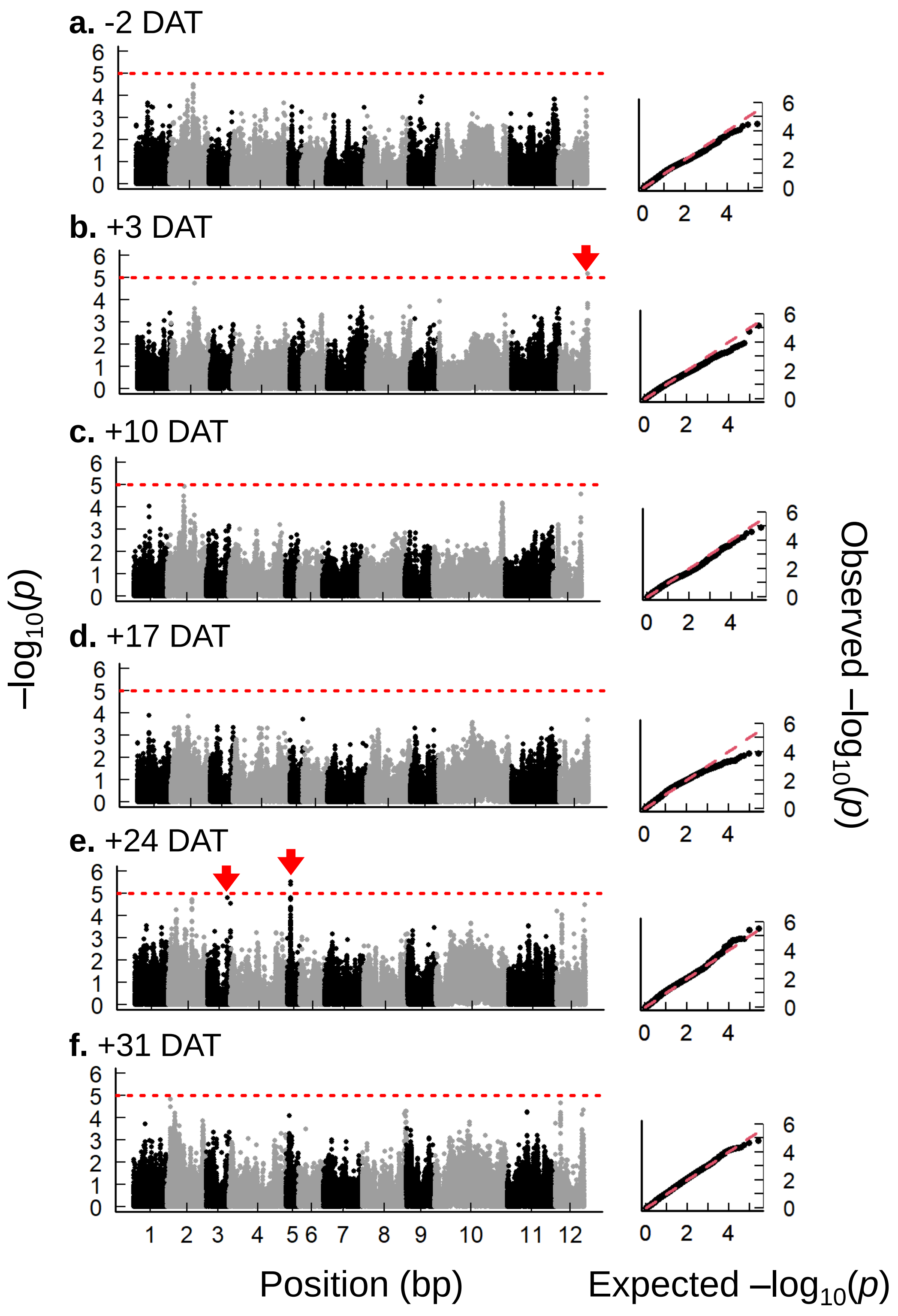

Figure S3.

Manhattan plots and quantile-quantile (Q-Q) plots of plant length (PL) under deep-water (DW) in 2021.

(**a**) -2 days after treatment (DAT): (**b**) +3 DAT: (**c**) +10 DAT: (**d**) +17 DAT: (**e**) +24 DAT: (**f**) +31 DAT in 2021. The x-axis indicates the SNPs or indels that physically mapped on each chromosome. The red arrows indicate the candidate region. The red dashed lines indicate the threshold lines ($-\log_{10}(p) = 5$) set in this study.

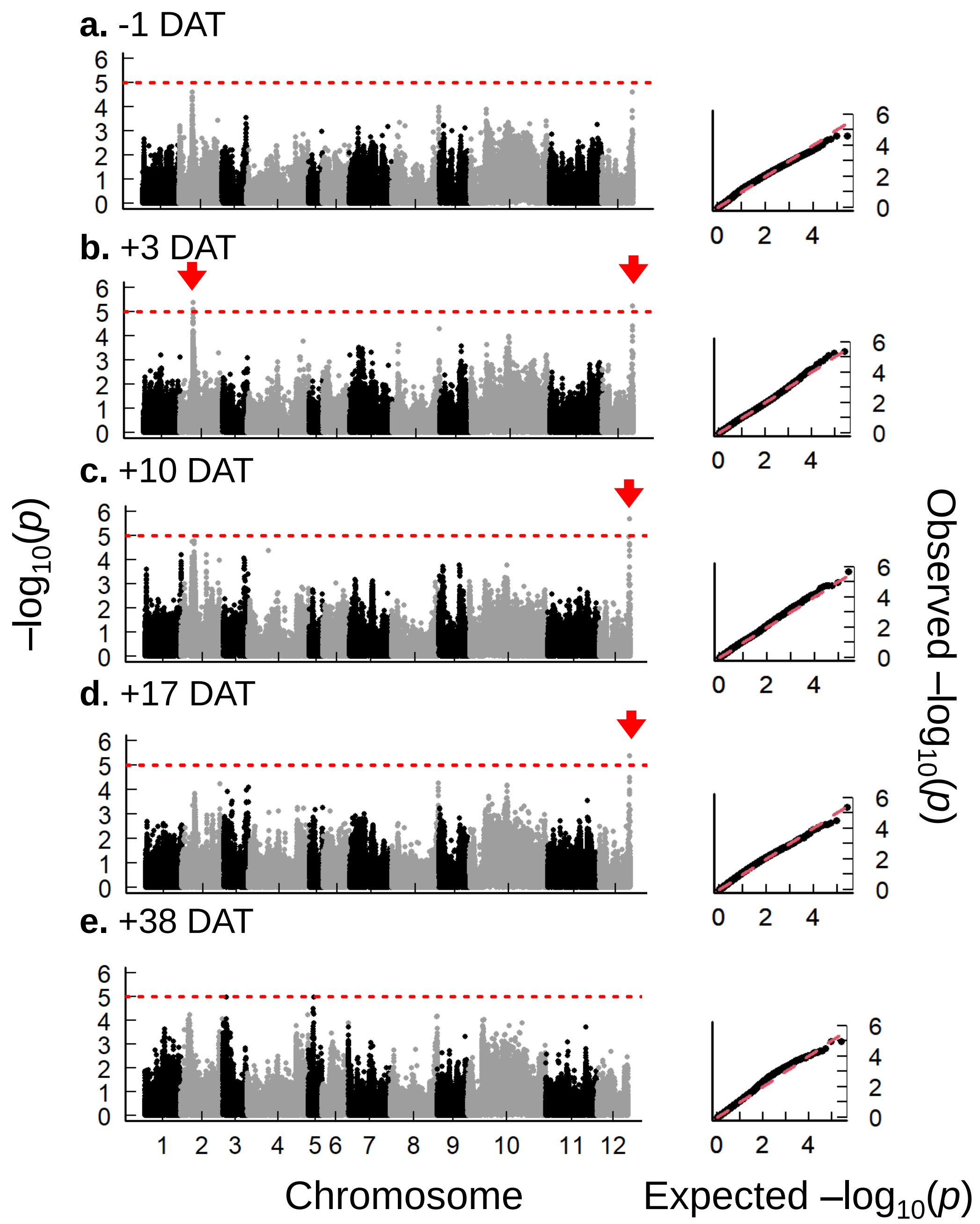

Figure S4.

Manhattan plots and quantile-quantile (Q-Q) plots of plant length (PL) under shallow-water (SW) in 2020.

(**a**) -1 days after treatment (DAT): (**b**) +3 DAT: (**c**) +10 DAT: (**d**) +17 DAT: (**e**) +38 DAT.

The x-axis indicates the SNPs or indels that physically mapped on each chromosome. The red arrows indicate the candidate region. The red dashed lines indicate the threshold lines ($-\log_{10}(p) = 5$) set in this study.

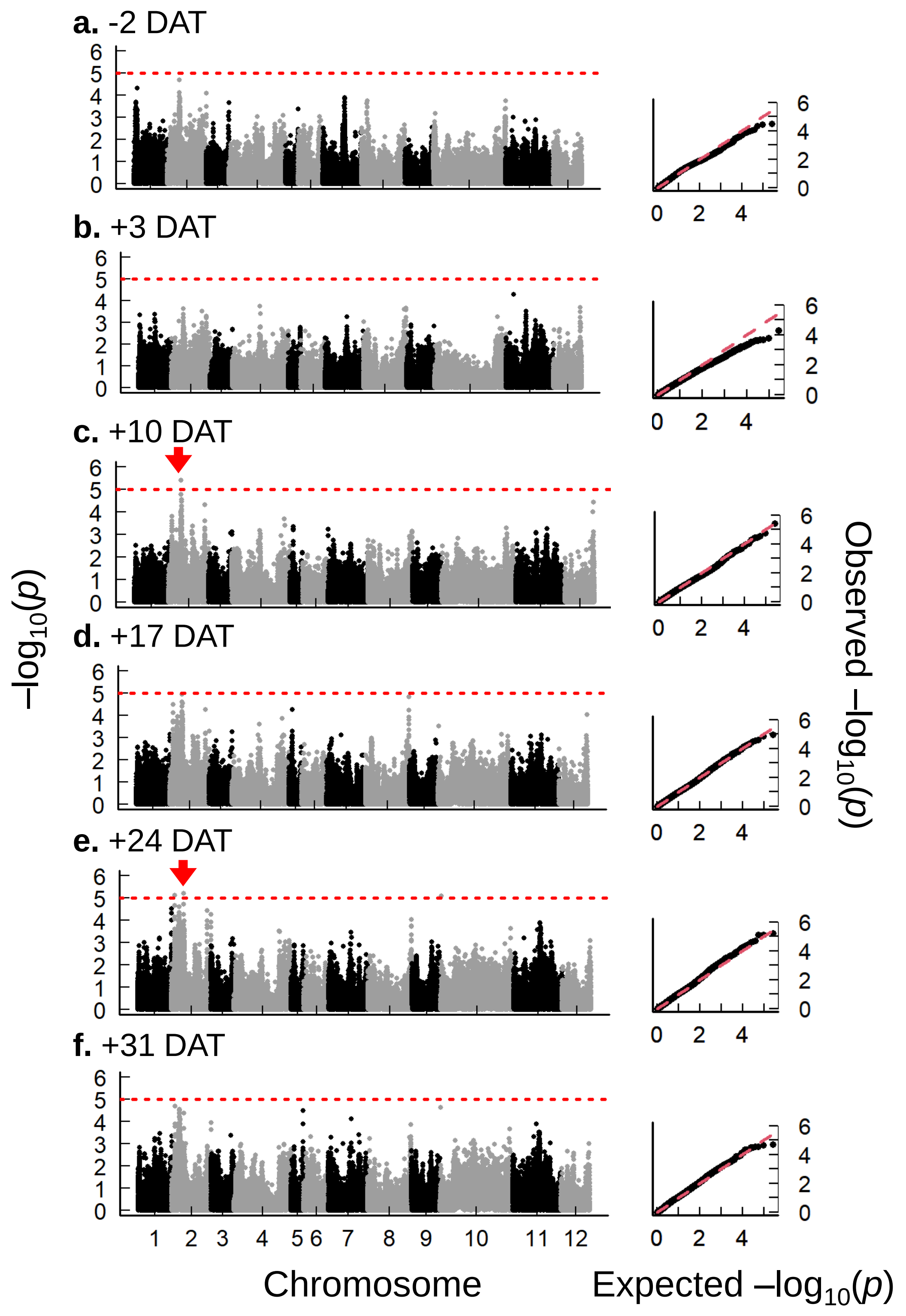

Figure S5.

Manhattan plots and quantile-quantile (Q-Q) plots of plant length (PL) under shallow-water (SW) in 2021.

(**a**) -2 days after treatment (DAT): (**b**) +3 DAT: (**c**) +10 DAT: (**d**) +17 DAT: (**e**) +24 DAT: (**f**) +31 DAT. The x-axis indicates the SNPs or indels that physically mapped on each chromosome. The red arrows indicate the candidate region. The red dashed lines indicate the threshold lines ($-\log_{10}(p) = 5$) set in this study.

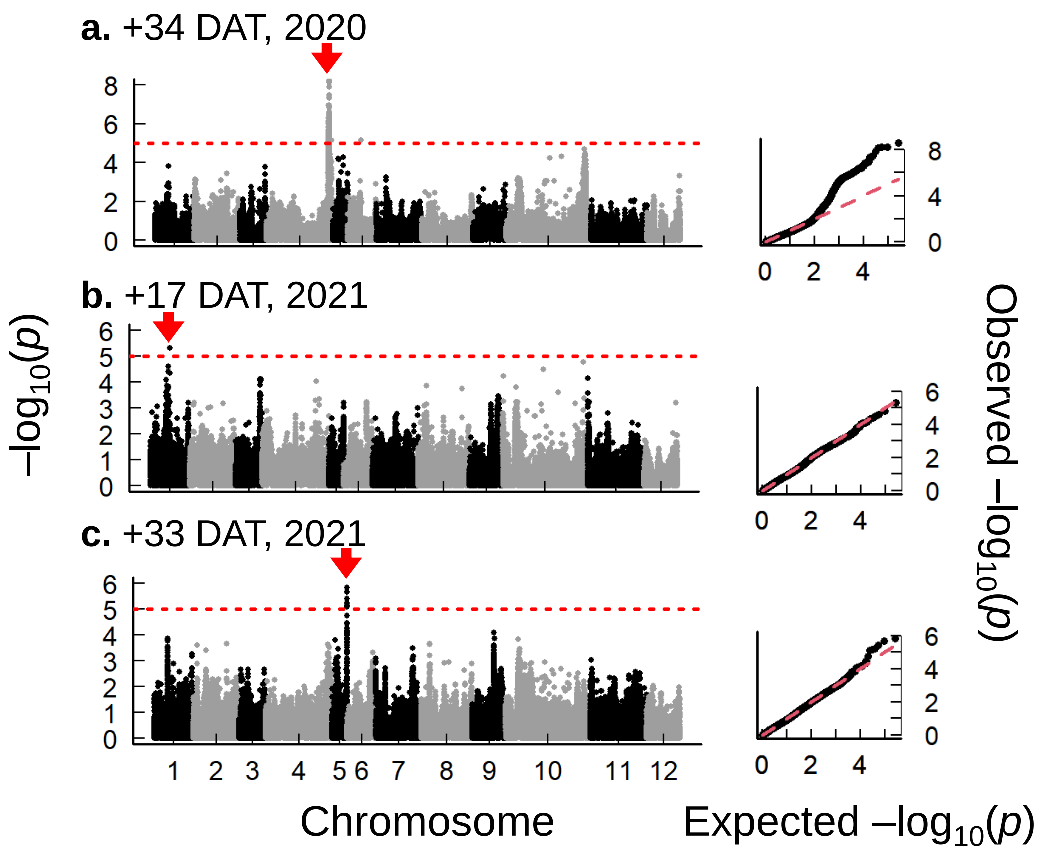

Figure S6.
Manhattan plots and quantile-quantile (Q-Q) plots of tiller number (TN) under shallow-water (SW).
(**a**) +34 days after treatment (DAT) in 2020: (**b**) +17 DAT in 2021: (**c**) +33 DAT in 2021.
The x-axis indicates the SNPs or indels that physically mapped on each chromosome. The red arrows indicates the candidate region. The red dashed lines indicate the threshold lines ($-\log_{10}(p) = 5$) set in this study.

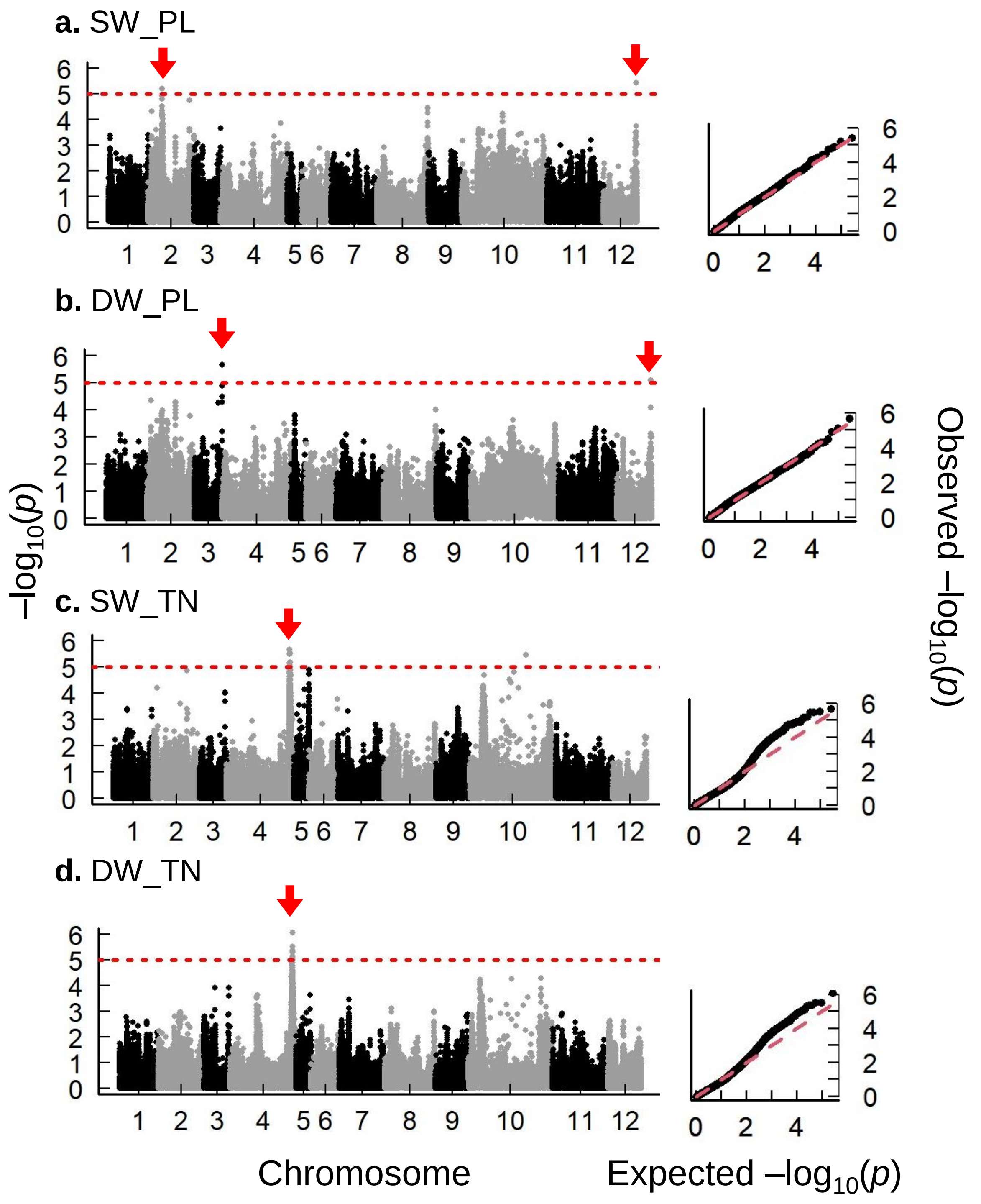

Figure S7.

Manhattan plots and quantile-quantile (Q-Q) plots of plant length (PL) and tiller number (TN) analyzed by Joint-GWAS.

(**a**) PL under shallow-water (SW): (**b**) PL under deep-water (DW). (**c**) TN under SW: (**d**) TN under DW.

The x-axis indicates the SNPs or indels that physically mapped on each chromosome. The red arrows indicates the candidate region. The red dashed lines indicate the threshold lines ($-\log_{10}(p) = 5$) set in this study.

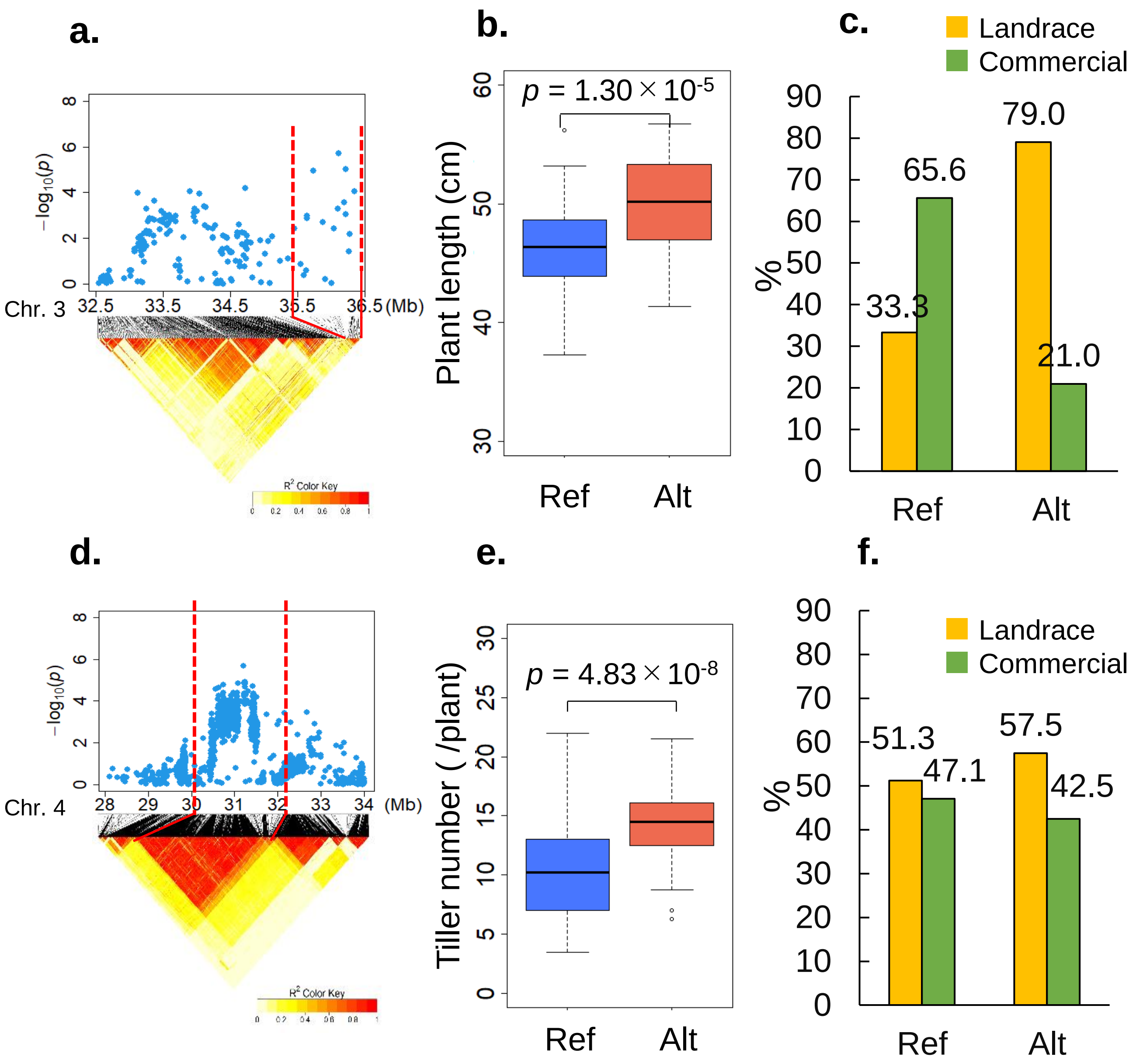

Figure S8.

Detailed analysis of *qPL3* and *qTN4*.

a,d: The enlarged view of the candidate region and LD heat block of *qPL3* (**a**) and *qTN4* (**d**).

b,e: Plant length at +10 days after treatment (DAT) for the indicated genotype of *LOC_Os03g64200* (**b**). TN for the indicated genotype of *LOC_Os04g52479* (**e**). Differences between varieties with reference genotype (ref; colored by blue) and alternative genotype (alt; colored by red) were analyzed by Welch's t-test.

c,f: The allele frequency between landraces (yellow) and commercials (green) at the peak of *qPL3* (**c**) and *qTN4* (**f**).

ATATATATAGCTACTAGCTAGCTTGTTTGCATATATAGATCAGCTTAGATGCATTACAATTAATTGTATGCATAAATGGTAATATCGTTC 90

CAACTAAATAGCTGACATCAGCATGGTATTAAATTTGGTACCTACGGAGTAGTATCATGTTTGATACCAGAGACACTAGCCAATTAATCC 180

CGTGTGGTTCCGGTGCACCGCGGCTTCTGAGAGGAGCTCGGGGAGGCATGTGTGGCTGCTGTTGTGGTGCCGACGGCGAGGGGCAAGTTC 270

TTGTCGTCTCCGGTGCACGGGCACGACAACGAAGCTCGTGCTCCTGTGGGTGCCAGAACCGCCGAGCAAGGTGTTCGTTGCGGCCACCTC 360

GATCGCCAATATCAACAAGCAGCTCATCTTCTCCATCGCGTGCCAAGGTTGAAGCGCCTCGATCGGTAGGTGGATCGCTCGCTGCTGAAG 450

GAGGATATGATGGTTGTGACAGGCCGGCATGCTGGCGGCCGGCACCGACGACGCTGGTGGCCACGAAGGTAGATGTCGACTGAGCAGGCT 540

CCTTGCCATCGCCCTTCTCCAATAAGCGGCACACCTGCCTAGTCCCTCCTTCCTATCCATGTCTACCTTACACATTGTGTGCTCCGGCCA 630

GCTCGTGAGCCAGAACGAGCTGGCTGGAGCTTTGCTTCCGAGCCGAGCCGAACTCCACTTTTTGCTCATTTAGATAACGAGCCGAGTCAA 720

GCCAGCTCGTTATCTAAATGAGCTGGAGCGACATATACCCCTATCTGTATACTGAAGGTATCATATCCGATACTTAGGGTATCATACTGA 810

CATCATCATGATACCTAAATACTAGATTTGATACTTGTGGGGTATCATGTTTGGTACCAGAGGTATATATACCAGATCTGATACCTATTT 900

GGTATCGTGCTGACATCATACGTTATTCTATTGAAACGGTATACCACTACGTAGTCTCACTCAATAATATACTATATATGTACGGGTAGG 990

ATGGAAGGTACAATGCAATCTTTGCGTAAGAGAAGAAGAGCGATTTGGTGGGTAGCTAGTTATCTATCTATCTCATGCTGTTTTGTCTTG 1080

ATTGCTGGACCACATGCATCAACATGATGTGGCCTGGCTCTGGCTGTAATAGTTCTATGGAAGAGAGAGCCAGAGCCAGCAGCATCAGCA 1170

TGCATGATCAAGGAGAGAGAGAGAGAGAGAGAGAGAGATGCATGAAGACGTACTAGCTAGCTGTGTGTCGTCTTCTAGCTCGATCGTGTTCG 1260

CTGATTCTAGATTAGGATGTTAATTGCGTGGTTGACATGCGTACTGCCAAAACCATCCATCGGTACGCCAGATTAATTAACAATCTCTAA 1350

TTCCAAGATCGATCTACGCTGTAGCATGCAGTACATTTTATATATGTATAGTTAACTTATTATATCTGACATATATTTGTTTGGGAGCCA 1440

ACAGACAAAATACATGAAAGACAAATTAGTTGAGGCTAGCTAGCTGCGCTGCTGCCTAGCTCTATGTAATGGATCGATCGATCATATGGC 1530

ACCAGCTAGCAGCTATCTTCGTCCTCATTAAGTTTAAACCAACTGCTTGATATATATGCATGCATGGAGCGTAGATATATATATATATAT 1620

ATATATATATAAAAGCTGTTGCGATGGTTGAGACGATTCAAACACACACAGAAAAAGCAAAAAAGTATCGAGCAATAATCCATATATGAG 1710

TAAATATTTAAAATAAATCCAGCGTAGATGCTAAGGGAACTCGAGTCCTCCTATCCCGCTAGATCACCATTTAATTAAGGAGGAAGGGAG 1800

GGTAGAAGAAGCGGGAGAAGAGGAAGAGTAGGAAGGGTAGAAAGAAAAATAAGAGTTCCCCTCTAATTTAATTCTGGATATGCTCCTTAA 1890

TGCAACAAAAGAATAATATTCTCTAGAGATAGAAGGCCAAATTATTAATAGAGAGAATCCCTTATATACCACTCTAAATTAACCGGTTCC 1980

CTTATATGCCACTTCAAATTGGCTTCTCTCTTCTATGCCACCAGTTCAAGTTTGTTATCCCTTTTATGCCATTGCCGTCACTCCACCGTC 2070

AGTTGACCGTTAACTTCATAGTAAAAAGACGCATTTGCCCTTGAGAGTTGGTATACAACCAATGATAGGTATTTTAATACTAATATAGCT 2160

ACTGGATACAACATATGAAAATTGATTCTTTTTTGCATTATATGCAATTTTAGCCATCACCATCACAAATAAATTTTCAATATAAGCATG 2250

AAACATACAGTTTCCAACAATATTTTTAATACAAAGAGCATTATTCATTGGTTTGGACAAAATTTTGTTCACTCAAAACGGATTTAAAAT 2340

AAAATAGTTATGATTTTTTGAAGTTTACACATTTTTTTAGTTGAGAGGGCATATTGGTCATTTACGAAAAAAACTAACATGCGACTAACA 2430

GTCAACTAATGCTGCAGTGCTGAGGATGGCATAAAAGGGTAGAAAAACTTAACCAGTGATATATAAGGGAGAAGCCAATTTTAATTGGCG 2520

TGTAAGGGAACTGGTCAATTTCTAGTAGCATATAAGAGATTCTCTCTTATTAATATGACAGTTCTCAGCTTGTGTATGGCCTCTCAAAGG 2610

CCATGCTTTTATATATTTTCACCCTATCAAAACTCTTTTCATATTAGACTACCTCAGTTTATGGTCAATTATATAGATCCTATTCGAAGC 2700

CAACCCTCAATCCATTTTTTTTCGCAAATTTGTGTGAAGCTGGATTTAAAATATATAATTCTTGAATTAAATATAAACAACTCTCTTATTT 2790

CTATTAGCCTGTTATAATTTATATACAACTATATCTATTAATTTATAATCTAGACCAACCACAATCTAGGAATGTAAATCTTTCTGTTTT 2880

TCTTATTAATGTAGTAATAATTTCTAGGTTTTTGTGAACGCGCAATAATTTCTAGGTCTCTCTAGAGTAACATGACCTTATTGCGGAGTT 2970

GTTTGTCCAGTCATCAATCTGGTGGTGGCACAGGCGCTGGGGTGTTTTGTAGTTTCTACTTGAGTAAATTGCATAAACGTGAATTTCACG 3060

Figure S9. continued

```
TTTTAAAAATCAAACATGTGAAGTGGTTGTCTAAATCTCGACATAGCGTCATGTCGCAGTACAACAACACTTCTGCAACGCCAGCCAAAG   3150

AAAAAAAAACTTGCATTTAGATAACCCTTGCATGTCCAAGATAAAATTGGCCCAAGTGAAAAACTCAGAACTACATAAATAATTTTCGCA   3240

AATGGCCAAAACTCATTTTTGGATGTGGTATTTTGACGTGTGGGCATCACAGGTGCACGTGAAAACCCATTTTCACATATGGATCTGTTG   3330

AGGAGGCTGCTTGCAAAACTGATTTTTGCAGGCAGGCAAGTAAGGACTTTCGACTAGAACGATTTTTTTTTTTAAAAAAAAGAATAAACA   3420

CTACAGATCCAAGACCCTATAGCTAGGGTAAGCCGCATGTGGCCGCGGCCGTGTGGTCCGTTGGCTGGGTACAAAGAGCAGAGTGGGCTA   3510

AGCAAATACCGGGTTGTGGCACCATCCCTTTCACATTCACTCGCTCTTGATATCTTTCTCTCTCATGGAAAAGAAGAGATAAGTAATTTT   3600

AATTGATGCCGGGATAGAGAGAGAGAGAGAGTTAAGAAGGTAGCTAGGGGAGAGAGCGAGGTTGATGCCGTGATCGATCGATCGATCTGT   3690

TGGCGCAGCGTGTATATAAGGGCGGGAAGGGGAGTGAGAGAGAGCAGCAGCTAGCTAGCCGCGGTCGGTCGATCCAGCTGCTGGGGATGA   3780

GTACTTAGTTAGCTCGGAGCTAGCTACTAATGGATGATATACTTATGCTAGTTAGTTAAATACAGTTATTAGTTAGTTGTAGGTTGCATC   3870

TATCATATCTCCATCGGTTAATTAATTGATTGATAGCTAGATTATCAACAATTAATGAGCATGGTGGTGCAGCAGGAGCAGGAGGTGGTG
                                                              M  S  M  V  V  Q  Q  E  Q  E  V  V   3960
TTCGACGCGGCGGTGCTGAGCGGGCAGACGGAGATCCCGTCGCAGTTCATATGGCCGGCGGAGGAGAGCCCCGGGTCGGTGGCGGTGGAG
F  D  A  A  V  L  S  G  Q  T  E  I  P  S  Q  F  I  W  P  A  E  E  S  P  G  S  V  A  V  E   4050
GAGCTGGAGGTGGCGCTGATCGACGTGGGGGCGGGGGCGGAGAGGTCGTCGGTGGTCCGGCAGGTGGGGGAGGCGTGCGAGAGGCACGGC
E  L  E  V  A  L  I  D  V  G  A  G  A  E  R  S  S  V  V  R  Q  V  G  E  A  C  E  R  H  G   4140
TTCTTCCTGGTGGTTAACCACGGCATCGAGGCGGCGCTGCTGGAGGAGGCGCACCGGTGCATGGACGCCTTCTTCACGCTGCCGCTGGGG
F  F  L  V  V  N  H  G  I  E  A  A  L  L  E  E  A  H  R  C  M  D  A  F  F  T  L  P  L  G   4230
GAGAAGCAGCGGGCGCAGCGGCGCGCGGGGGAGAGCTGCGGCTACGCCAGCAGCTTCACGGGGCGCTTCGCGTCCAAGCTGCCGTGGAAG
E  K  Q  R  A  Q  R  R  A  G  E  S  C  G  Y  A  S  S  F  T  G  R  F  A  S  K  L  P  W  K   4320
GAGACGCTGTCGTTCCGGTACTCATCGGCTGGAGATGAAGAGGGCGAGGAGGGCGTGGGTGAGTACCTGGTGCGGAAGCTCGGGGCGGAG
E  T  L  S  F  R  Y  S  S  A  G  D  E  E  G  E  E  G  V  G  E  Y  L  V  R  K  L  G  A  E   4410
CACGGGCGGCGGCTGGGCGAGGTGTACTCGCGCTACTGCCACGAGATGAGCCGCCTGTCGCTGGAGCTGATGGAGGTGCTCGGGGAGAGC
H  G  R  R  L  G  E  V  Y  S  R  Y  C  H  E  M  S  R  L  S  L  E  L  M  E  V  L  G  E  S   4500
CTGGGCATCGTCGGAGACCGGCGCCACTACTTCCGGCGATTCTTCCAGCGCAACGACTCCATCATGCGCCTCAACTACTACCCGGCGTGC
L  G  I  V  G  D  R  R  H  Y  F  R  R  F  F  Q  R  N  D  S  I  M  R  L  N  Y  Y  P  A  C   4590
CAGAGGCCACTCGACACGCTGGGCACCGGTCCGCACTGCGACCCCACCTCGCTCACCATCCTCCACCAGGACCACGTCGGCGGCCTGGAG
Q  R  P  L  D  T  L  G  T  G  P  H  C  D  P  T  S  L  T  I  L  H  Q  D  H  V  G  G  L  E   4680
GTGTGGGCGGAGGGGCGGTGGCGCGCCATCCGCCCTCGCCCCGGGGCGCTCGTCGTCAACGTCGGCGACACCTTCATGGCGCTCTCCAAC
V  W  A  E  G  R  W  R  A  I  R  P  R  P  G  A  L  V  V  N  V  G  D  T  F  M  A  L  S  N   4770
GCCAGGTACCGCAGCTGCCTGCACCGGGCGGTCGTCAACAGCACGGCGCCTCGCCGCTCGCTGGCCTTCTTCCTCTGCCCGGAGATGGAC
A  R  Y  R  S  C  L  H  R  A  V  V  N  S  T  A  P  R  R  S  L  A  F  F  L  C  P  E  M  D   4860
ACGGTGGTGCGCCCGCCGGAGGAGCTGGTCGACGACCACCACCCGAGGGTGTACCCGGACTTCACGTGGCGGGCGCTGCTGGACTTCACG
T  V  V  R  P  P  E  E  L  V  D  D  H  H  P  R  V  Y  P  D  F  T  W  R  A  L  L  D  F  T   4950
CAGCGCCACTACAGGGCCGACATGCGCACGCTTCAGGCCTTCTCCGACTGGCTTAATCATCATCGTCACCTGCAACCAACAATATACTCC
Q  R  H  Y  R  A  D  M  R  T  L  Q  A  F  S  D  W  L  N  H  H  R  H  L  Q  P  T  I  Y  S   5040
TAGCTCCTAGTCCTAGCTATATACTCCTATTATCCATCCATCCATCCATCTTACACTACTATACCATTAGCATCGATCGATCATCCATTA
 *                                                                                          5130

ATTAATTAATTAATTACTAGTTCCGGCTTAGATATATATCTGGCGATTATTTCAGTTCCTAGCTACTCCTACATGCATGCTTTGCTTAAT   5220

TAGATCTATCTATCTAATCTATCCCGGCCGGCCTGTTTTAATTCCATATATCATTTGGTTTGCACGTACCCATCTATGATCTATATATAC   5310

ATGCATGTCGACTATTGTTGGTCGTACGATATTATATTATATATAGATGTAATACATATGTTGAAAATATATTGATTTCTTCTTGCTGTA   5400

GGAGTAATTAATTACTCCAGTTGTTGCTGTCACTCGATCAATCCATGGGATATGGGGATCGATCGGACGATATGCAGGAAGCTCAAGGAC   5490

GATATGAATGATGCATGCATGTGTAAATGTGTTGTTGTGTTTGGTTTCTCTTTTGTCGTTTCCCCTGCCCGTCCGTTCGTCCCTCAGCTT   5580

TTTACCTATATACTATAGCTACCTACACAAACAAACAAACAAAGAAAGAGAGACATATGAATCGATTACTAGTAACATCGATCTATCAAT   5670

GGTTGCTTCAATTTCTACTTCTCAAAAGAA                                                               5700
```

Figure S9. Nucleotide and amino acid sequences of 5.7 kb region of *OsGA20ox1*.

None of the nucleotide sequence had any mutation among the four varieties listed in Table S2.

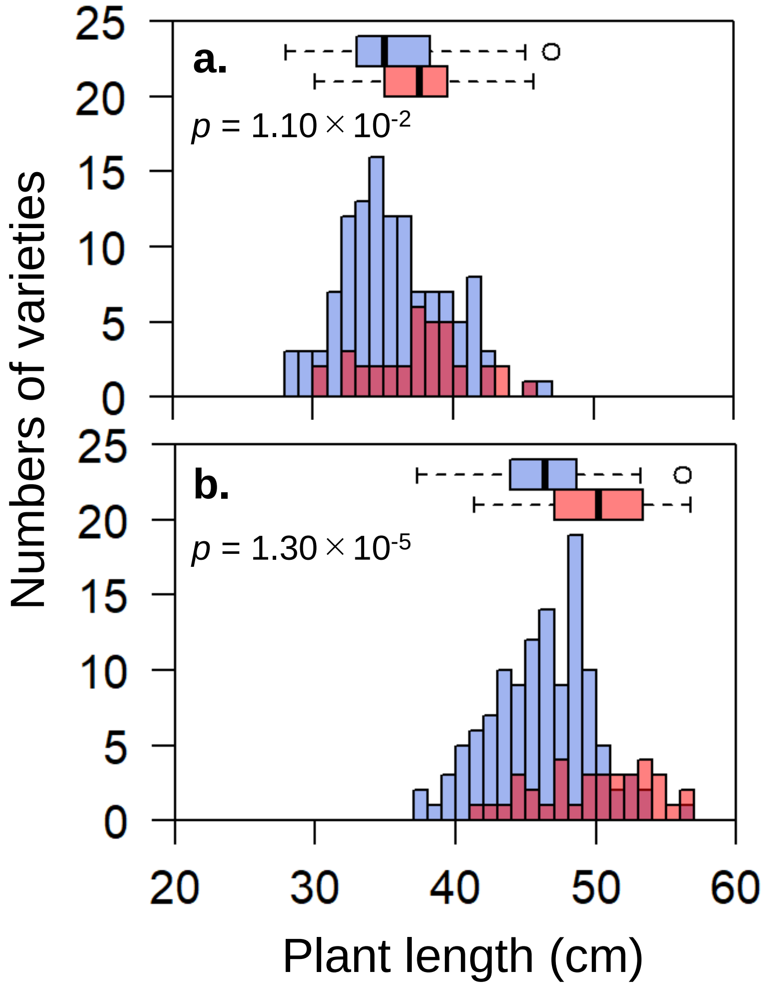

Figure S10.
Histogram of plant length (PL) at +10 days after treatment (DAT) in 2020.
(**a**) Data in shallow-water (SW). (**b**) Data in deep-water (DW).
Blue and red bars indicate the results of varieties with reference and alternative genotype of *qPL3*, respectively.
